# Inhibitors of the PI3K/mTOR pathway prevent STAT5 phosphorylation in *JAK2V617F* mutated cells through PP2A/CIP2A axis

**DOI:** 10.18632/oncotarget.18073

**Published:** 2017-05-22

**Authors:** Niccolò Bartalucci, Laura Calabresi, Manjola Balliu, Serena Martinelli, Maria Caterina Rossi, Jean Luc Villeval, Francesco Annunziato, Paola Guglielmelli, Alessandro M. Vannucchi

**Affiliations:** ^1^ CRIMM, Centro di Ricerca e Innovazione per le Malattie Mieloproliferative, Azienda Ospedaliera Universitaria Careggi, Florence, Italy; ^2^ Department of Experimental and Clinical Medicine, University of Florence, Florence, Italy; ^3^ DENOTHE Excellence Center, Florence, Italy; ^4^ INSERM, Unité Mixte de Recherche (UMR) 1170, Institut Gustave Roussy, Villejuif, France

**Keywords:** STAT5, phosphorylation, MPN, ruxolitinib, PI3K/mTOR inhibitors

## Abstract

Inhibition of the constitutively activated JAK/STAT pathway in JAK2V617F mutated cells by the JAK1/JAK2 inhibitor ruxolitinib resulted in clinical benefits in patients with myeloproliferative neoplasms. However, evidence of disease-modifying effects remains scanty; furthermore, some patients do not respond adequately to ruxolitinib, or have transient responses, thus novel treatment strategies are needed. Here we demonstrate that ruxolitinib causes incomplete inhibition of STAT5 in *JAK2V617F* mutated cells due to persistence of phosphorylated serine residues of STAT5b, that conversely are targeted by PI3K and mTORC1 inhibitors. We found that PI3K/mTOR-dependent phosphorylation of STAT5b serine residues involves Protein Phosphatase 2A and its repressor CIP2A. The levels of CIP2A were found increased in cells harboring the JAK2V617F mutation, and we provide evidence of a correlation between clinical responses and the extent of CIP2A downregulation in myelofibrosis patients receiving the mTOR inhibitor RAD001 in a phase II clinical trial. To achieve maximal inhibition of STAT5 phosphorylation, we combined ruxolitinib with BKM120, a PI3K inhibitor, and RAD001, an mTOR inhibitor, obtaining improved efficacy in *JAK2V617F* mutated cell lines, primary patients’ cells, and *JAK2V617F* knock-in mice. These findings contribute to understanding the effectiveness of PI3K/mTOR inhibitors in MPN and argue for the rationale to develop combination clinical trials.

## INTRODUCTION

Clonal proliferation of hematopoietic progenitor cells and dysregulated production of blood cells of different lineages are the main characteristics of chronic myeloproliferative neoplasms (MPN) [1]. MPN include Essential Thrombocythemia (ET), Polycythemia Vera (PV) and Primary Myelofibrosis (PMF). Following the description of the *JAK2*V617F mutation [2, 3], additional somatic driver mutations were discovered in *JAK2* wild-type patients, including *JAK2* exon12 mutations in PV [4] and thrombopoietin receptor (*MPL*) [5, 6] and calreticulin (*CALR*) mutations in ET and PMF [7, 8]. Additional myeloid-malignancies associated mutations involving epigenetic or spliceasome genes were described in 5% to 40% of MPN patients, and serve as prognostic variables [9, 10].

The JAK1 and JAK2 inhibitor ruxolitinib [11] is approved for patients with myelofibrosis [12] and with PV [13, 14] resistant or intolerant to hydroxyurea. However, ruxolitinib, as well as other JAK2 inhibitors under development, is not selective for the mutated JAK2 and inhibits the JAK/STAT signaling also in JAK2 wild-type cells. Although ruxolitinib reduces to some extent the mutated allele burden after long-term treatment, only few patients eventually achieve complete molecular response [15]. The lack of a clear disease-modifying effect of ruxolitinib [16, 17] might be ascribed to an underlying mutation complexity of clonal hematopoietic progenitors and/or to hyperactivated signaling pathways other than JAK/STAT, including in particular the PI3K/mTOR cascade [18–21].

In normal cells, PI3K-dependent signals contribute to the regulation of cell survival and proliferation through the activation of downstream kinases such as Akt and the mammalian target of rapamycin, mTOR; the latter is found in the different complexes, mTORC1 and mTORC2, and in turn controls downstream effectors including the transcription factor 4e binding protein 1 (4eBP1) and p70 s6 kinase (p70s6K). In normal hematopoiesis, the PI3K/mTOR pathway is essential for erythroid and megakaryocytic differentiation [22]. The different key kinases of the PI3K/Akt pathway were found deregulated or hyperactivated [23] in solid and hematologic malignancies [24, 25]. This pathway is deregulated also in cells expressing the *JAK2*V617F mutation [3], particularly in megakaryocytes of MPN patients [26].

Evidences favoring the relevance of targeting PI3K/mTOR pathway in MPN derive from cellular and animal models [27, 28]; furthermore, in a phase I/II clinical trial in patients with myelofibrosis, the mTORC1 inhibitor RAD001 (Everolimus) demonstrated clinical effectiveness by reducing splenomegaly and improving constitutional symptoms [29]. Other studies demonstrated an added efficacy by combining inhibitors of the PI3K/mTOR and JAK/STAT pathway [30, 31]. However, the molecular basis of the functional crosstalk between the JAK/STAT and PI3K/mTOR pathway in *JAK2*V617F mutated cells remained poorly characterized. In this work, we show that signals originated by the activated JAK2 and PI3K pathway in *JAK2*V617F mutated cells specifically control the phosphorylation of different residues of STAT5. This might contribute to explain the persistence of activated STAT5 in cells exposed to JAK1/2 inhibitor ruxolitinib alone as well as the synergism exerted by combination of ruxolitinib with PI3K and mTOR inhibitors. Overall, these data provide mechanistic explanation for the involvement of PI3K/mTOR signaling in cells harboring the *JAK2*V617F mutation and might be translationally relevant for designing innovative strategies in MPN.

## RESULTS

### STAT5 is differentially phosphorylated by activated JAK2 and PI3K/mTOR pathway in JAK2V617F mutated cells

**Figure 1:**
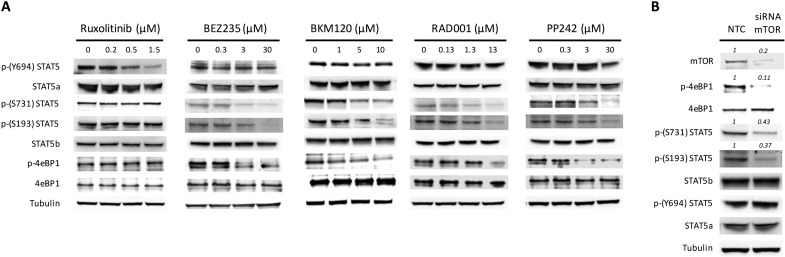
PI3K/mTOR inhibitors modulate phosphorylation pattern of STAT5 (**A**) pattern of phosphorylation of tyrosine and serine residues on STAT5a and STAT5b, respectively, and the effects of the JAK1 and JAK2 inhibitor ruxolitinib and of different inhibitors of the PI3K/mTOR pathway. BKM120 is a PI3K inhibitor, BEZ235 is a dual PI3K/mTOR inhibitor, RAD001 and PP242 are inhibitors of mTORC1 and mTORC1/2, respectively. SET2 cells were exposed for 24 h to increasing concentrations of the drugs and cell extracts were processed by western blotting analysis with indicated antibodies. A representative experiment of at least four. (**B**) Effects of mTOR silencing by specific siRNA on the levels of phosphorylated tyrosine and serine residues of STAT5a/b in SET2 cells. The mTORC1 target phospho-4eBP1 was used as a control of functional inhibition of mTORC1 signaling. Tubulin was used as loading control. Densitometric analysis was performed using ImageJ software. Data are from one representative experiment out of three.

**Figure 2:**
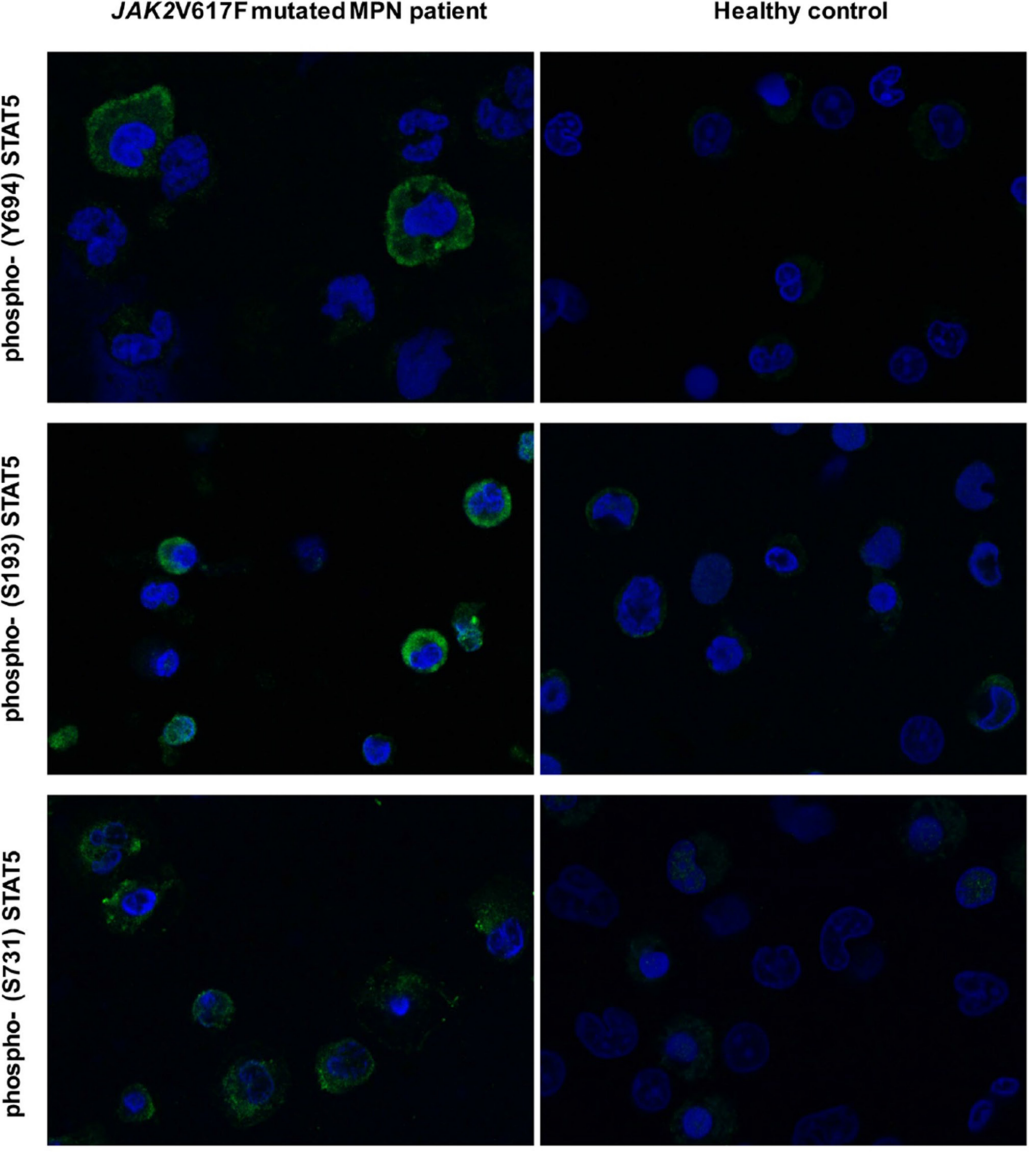
Autonomous phosphorylation of STAT5 residues Y694, S193 and S731 in untouched granulocytes from a *JAK2*V617F mutated patients with myelofibrosis, analyzed by confocal microscopy Control cells from a healthy donor are shown on the right. Cells were isolated by density gradient and probed with phospho-specific anti-STAT5 antibodies. Topro-3 was used as nucleus marker. Microscopic images were taken by a LSM 510 META Zeiss confocal microscope system, using 40X oil immersion lens, corresponding to a 400X magnification. For images analysis Zeiss Confocor 2 software was used. A representative experiment out of 5 is shown.

We previously reported that STAT5 phosphorylation in *JAK2*V617F mutated cells was down-regulated after exposure to drugs targeting the PI3K/mTOR signaling [27, 31]. In order to clarify the mechanism(s) by which inhibition of PI3K/mTOR signaling prevented STAT5 phosphorylation, we comparatively examined the pattern of phosphorylated STAT5a and STAT5b residues following treatment of the cells with the JAK1/2 inhibitor ruxolitinib, different PI3K/mTOR inhibitors (mTORC1 inhibitor RAD001, mTORC1/2 inhibitor PP242, and PI3K/mTORC1/2 inhibitor BEZ235) and the pan PI3K inhibitor BKM120 (Figure 1A). As expected, using *JAK2*V617F mutated SET2 cells, we found that ruxolitinib dose-dependently reduced phosphorylated STAT5 Tyrosine-694 (Y694) that, together with Tyrosine-699 (not shown), is the immediate target of activated JAK2 in human STAT5a and STAT5b, respectively. Conversely, none of the PI3K and/or mTOR inhibitors tested were able to affect the phosphorylation of Y694. However, all the PI3K/mTOR inhibitors caused a dose-dependent reduction of the phosphorylation of Serine-731 (S731) and Serine-193 (S193) residues of STAT5b; of note, these serine residues on STAT5b were not affected by exposure to ruxolitinib. Since RAD001, an mTORC1 complex inhibitor, reduced phosphorylation of S731 and S193 at levels comparable to those obtained with BEZ235 and BKM120, that are PI3K inhibitors, we interpreted these findings as indicating that phosphorylation of serine residues of STAT5b is a RAD001 sensitive, mTORC1-dependent phenomenon. To support the hypothesis, we performed siRNA-mediated mTOR silencing in SET2 cells (Figure 1B). The expression levels of mTOR and its target phospho-4eBP1 were almost completely abolished at 24 hours following siRNA transfection, confirming effective silencing, and were accompanied by marked reduction of phosphorylated S731 and S193 residues of STAT5b, thereby excluding off-target effects of PI3K/mTOR inhibitors. Conversely, the levels of phosphorylated Y694 in STAT5a did not change appreciably after siRNAs treatment. Finally, we assessed the phosphorylated status of STAT5 tyrosine and serine residues in peripheral blood granulocytes of *JAK2*V617F mutated MPN patients using confocal microscopy (Figure 2). We found that phosphorylated Y694 as well as S193 and S731 residues were clearly marked in patients’ cells as compared to control cells. Overall, these findings indicate that in cells expressing the *JAK2*V617F mutation, STAT5 is phosphorylated on both tyrosine and serine residues as the result of JAK2- and PI3K/mTOR-dependent mechanisms, respectively.

### STAT5 de-phosphorylation induced by PI3K/mTOR inhibitors is mediated by Phosphatase-2a and its inhibitor CIP2A

**Figure 3:**
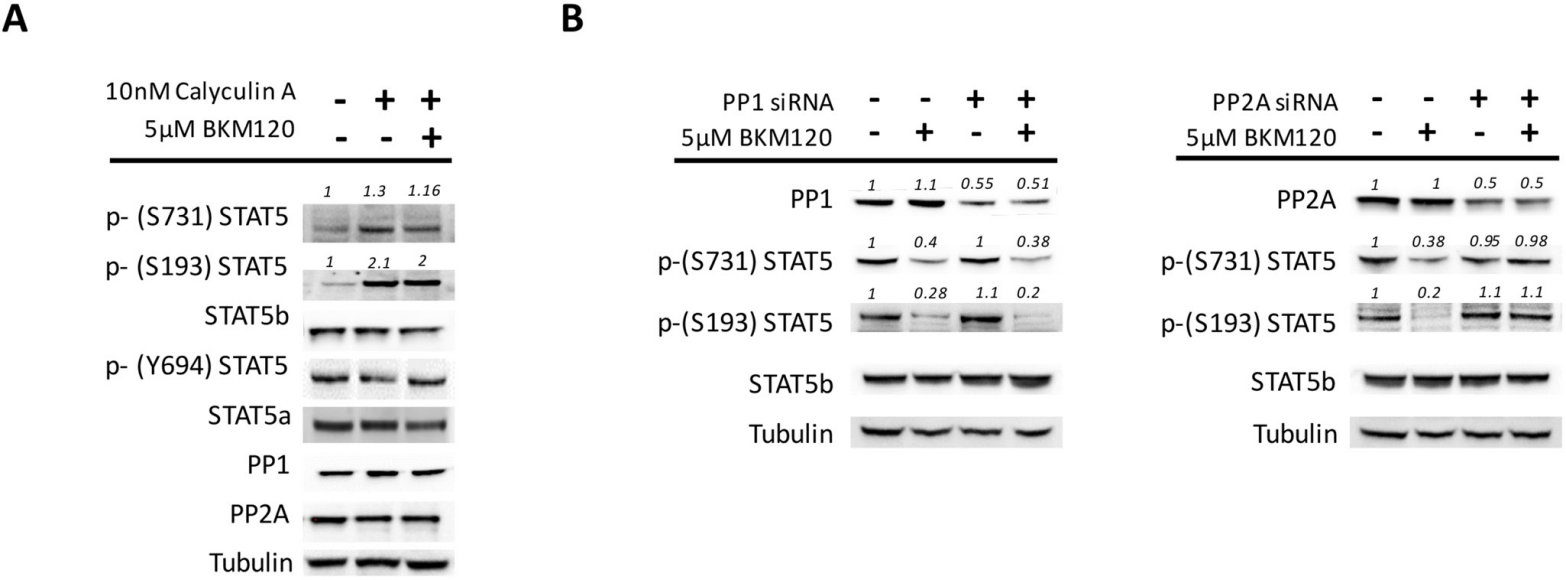
PP2A is involved in the de-phosphorylation of STAT5 serine residues in *JAK2*V617F mutated cells (**A**) SET2 cells were pre-treated for 2 hours with the chemical phosphatase inhibitor Calyculin A (CA; 10 nM) before the addition of 5μM BKM120 for 24 hours. CA exposure resulted in increased phosphorylation of STAT5b serine residues and prevented their BKM120-induced de-phosphorylation, as opposite to STAT5a Y694 residue that remained unchanged. (**B**) Specific siRNAs were used to silence, although only partially, either PP1 or PP2A to dissect their unique role in the BKM120-induced de-phosphorylation of S731 and S193 of STAT5b. Tubulin was used as loading control. One of 5 different experiments is shown. Densitometric analysis was performed using ImageJ software.

To address the mechanism(s) underlying STAT5 serine de-phosphorylation induced by PI3K/mTOR inhibitors in *JAK2*V617F mutated cells, we focused on protein phosphatases, that are the enzymes mainly responsible of protein de-phosphorylation [32–34], and their inhibitors. Owing that about 90% of cell phosphatases are constituted by phosphatase 1 (PP1) and 2 (PP2A) [35], we first used the chemical phosphatases inhibitor Calyculin A (CA). We found that CA exposure caused, respectively, a 1.3±0.2 and 2.1±0.4 fold increase of phosphorylated S731 and S193 residues of STAT5b (*P* < 0.05 for both *versus* untreated cells, mean±SD of 4 experiments), and also prevented their de-phosphorylation when BKM120 was added to the cells (Figure 3A). Notably, CA treatment did not affect the levels of phospho Y694. As expected by the mechanism of action of CA, the levels of PP1 and PP2A protein remained unchanged (Figure 3A). To assess the contribution of the different phosphatases, we silenced individually PP1 or PP2A with specific siRNAs before treating the cells with BKM120. Only PP2A silencing was capable to effectively prevent the BKM120-induced de-phosphorylation of S731 and S193, while down-regulation of PP1 had no effects on serine phosphorylation levels (Figure 3B). These changes could be reproduced in multiple experiments (*n* = 5) even though we succeeded in inducing only a 50–60% down-regulation of phosphatase expression levels (in spite of different sets of siRNAs employed, not shown in detail). Taken together, these data illustrate a prominent role of PP2A in the de-phosphorylation of STAT5b serine residues induced by BKM120, and indirectly support a defective PP2A activity in cells harboring the *JAK2*V617F mutation.

**Figure 4:**
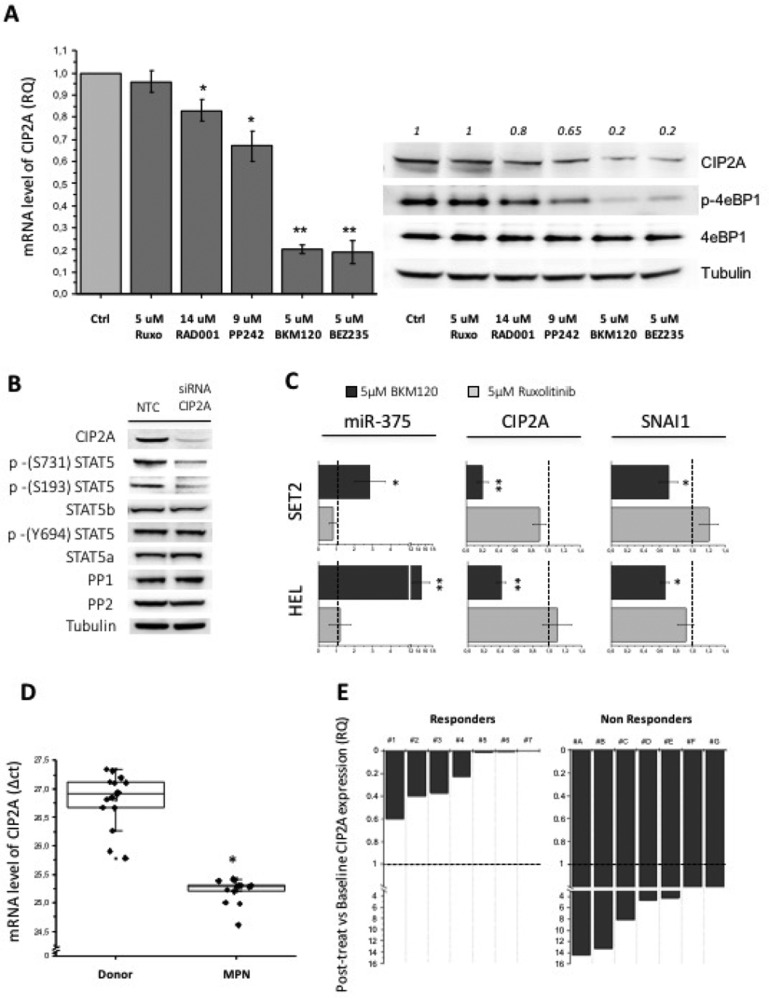
The expression of the PP2A inhibitor CIP2A affects the phosphorylation pattern of STAT5 serine residues (**A**) PI3K/mTOR inhibitors treatment of SET2 cells for 24 h induces a various degree of CIP2A mRNA downregulation (ranging from -20% to –80% *vs* control) as assessed by qRT-PCR (on the left) and western blotting (on the right) analysis. (**B**) CIP2A was silenced by specific siRNA, and the effects on the phosphorylation status of STAT5 S731, S193 and Y694 residues were monitored. (**C**) The levels of miR-375 and CIP2A and SNAI1 mRNAs were monitored by qRT-PCR in SET2 and HEL cells that had been exposed to BKM120 or ruxolitinib. (**D**) CIP2A mRNA levels were quantified by qRT-PCR in granulocytes isolated from the peripheral blood of 12 *JAK2*V617F mutated MPN patients and 15 healthy controls. (**E**) Correlation of changes of CIP2A mRNA levels with clinical response to treatment in 14 patients with myelofibrosis who had been enrolled in a phase II trial with the mTOR1 inhibitor RAD001 (everolimus). A qRT-PCR analysis was performed in peripheral blood samples collected after 3 months of treatment. All *P* values were determined by unpaired two-tailed Student’s T test and confirmed by BootstRatio test on fold change data (^*^P < 0.05, ^**^P < 0.01).

The activity of PP2A in the cells is mainly regulated by CIP2A (Cancerous Inhibitor of PP2A) and I2PP2a (Inhibitor 2 of Protein Phosphatase 2A) (also named SET) [36] through allosteric inhibition of phosphatase. We reasoned that reduced activity of PP2A, eventually leading to increased phosphorylation of STAT5 serine residues in *JAK2*V617F mutated cells, might result from excessive inhibition exerted by endogenous inhibitors. Therefore, we treated SET2 cells with different PI3K and mTOR inhibitors and with ruxolitinib and measured the levels of CIP2A and I2PP2A mRNA. Ruxolitinib did not change the baseline levels of CIP2A mRNA while PI3K inhibitor BKM120 and the double PI3K and mTOR inhibitor BEZ235 dramatically reduced CIP2A mRNA and protein levels (Figure 4A). The mTORC1 inhibitor Everolimus and the double mTORC1/2 inhibitor PP242 caused less impressive, yet statistically significant, down-regulation of CIP2A levels (Figure 4A). Of note, the extent of CIP2A down-regulation and the levels of phospho-4eBP1, the direct target of PI3K/mTOR, were congruent (Figure 4A). On the contrary, we found no appreciable change of I2PP2a mRNA levels with any of the inhibitors tested (Supplementary Figure 1), confirming the specific involvement of CIP2A in controlling PP2A activity in *JAK2*V617F mutated cells. Furthermore, we showed that siRNA-mediated inhibition of CIP2A mRNA resulted in marked de-phosphorylation of serines 731 and 193 of STAT5b, but not tyrosine 694; levels of STAT5a and b, PP1 and PP2A were unchanged (Figure 4B). It was reported that the tumor suppressor miR-375 represses CIP2A mRNA through multiple miRNA-mRNA interactions [37, 38], and it is in turn negatively regulated by SNAI1 (Snail) [39, 40]. Therefore, we wanted to ascertain whether the reduction of CIP2A levels determined by PI3K/mTOR inhibitors involved miR-375. We found that when BKM120 was added to SET2 and HEL cells, the levels of miR-375 increased markedly, and such an increase was associated with concurrently decrement of CIP2A and Snail. On the contrary, ruxolitinib did not affect Snail, miR-375 and CIP2A expression, therefore addressing specifically these effects to the PI3K/mTOR pathway (Figure 4C).

To clarify a possible role of CIP2A in the constitutive phosphorylation of serine residues of STAT5 in primary *JAK2*V617F mutated cells, we measured CIP2A mRNA levels in granulocytes of MPN patients (3 PV, 9 MF) and healthy controls (*n* = 15) (Figure 4D). We found that the levels of CIP2A mRNA were 3.28-fold higher (±1.2) in patients’ granulocytes compared to donors’ cells (*P* < 0.05), further supporting abnormally increased CIP2A levels as a mechanism for reduced PP2A activity in patients’ cells. The relevance of this observation was strengthened by the analysis of CIP2A mRNA levels in granulocytes from 14 patients with myelofibrosis who had received RAD001 in a phase II clinical trial [29]. The CIP2A mRNA levels were measured after 3 (±2) months of treatment and, after being normalized to pre-treatment levels, were correlated with clinical outcome according to the IWG-MRT criteria [41]. Interestingly, we observed that all 7 patients who had a clinical response to treatment (defined as reduction of splenomegaly and symptomatic improvement) showed overt down-regulation of CIP2A mRNA levels, ranging from -40% to -99.6% the baseline levels, while all the 7 patients who did not benefit from treatment (non responders) showed no changes or even an increase of CIP2A mRNA levels (Figure 4E). As a whole, results from these experiments point to exaggerated increase of CIP2A levels in *JAK2*V617F mutated cells as the main reason for defective PP2A activity, that might be responsible for deregulated STAT5b serine residues phosphorylation, and mechanistically associate such an increase with abnormal signaling originated by the PI3K/mTOR pathway.

### Concurrent inhibition of STAT5 phosphorylated tyrosine and serine residues produces synergism against JAK2V617F mutated cells

The findings of persistent STAT5b serine residues phosphorylation in *JAK2*V617F mutated cells exposed to ruxolitinib indicate incomplete inhibition of phospho-STAT5-mediated signaling. Tyrosine phosphorylation allows STAT5 to dissociate from the receptor complex and promotes the formation of hetero- or homodimers and their translocation to the nucleus to activate target genes transcription [42]. On the other hand, the phospho-serine sites located in a conserved Pro-Ser-Pro motif in the transactivation domain are required for maximum transcriptional activity of STAT5 [43, 44]. Therefore, we wanted to explore the hypothesis that complete de-phosphorylation of STAT5, involving also the serine residues in addition to the tyrosines that are inhibited by ruxolitinib, might result in more pronounced inhibition of *JAK2*V617F mutated cells. For these studies we used as relevant drugs the pan PI3K inhibitor BKM120, that is being tested in a clinical trial in association with ruxolitinib [45], and the mTORC1 complex inhibitor RAD001, that was already employed as single agent in myelofibrosis [29], and has been characterized in *in-vitro* models [27].

In a preliminary set of experiments, we evaluated the efficacy of BKM120 as single agent in *in-vitro* models of *JAK2*V617F mutated cells, by measuring changes in cell proliferation, cell cycle and apoptosis (Supplementary Figure 2), and by determining colony formation in cultures established with MPN hematopoietic progenitor cells (Supplementary Table 1 and Supplementary Figure 3); we also analyzed the effects of BKM120 on survival of mice injected with Ba/F3 *JAK2*V617F-Luc+ cells [31] (Supplementary Figure 4). Results from these experiments, that are detailed in the respective Figure legends, indicated that BMK120 was able to effectively inhibit the proliferation, and induce apoptosis, of *JAK2*V617F mutated mouse and human cell lines, potently inhibit the growth of erythropoietin-independent erythroid colonies from MPN patients, and reduce the dissemination of Ba/F3 *JAK2*V617F-Luc+ mutated cells in mice prolonging their survival.

**Figure 5:**
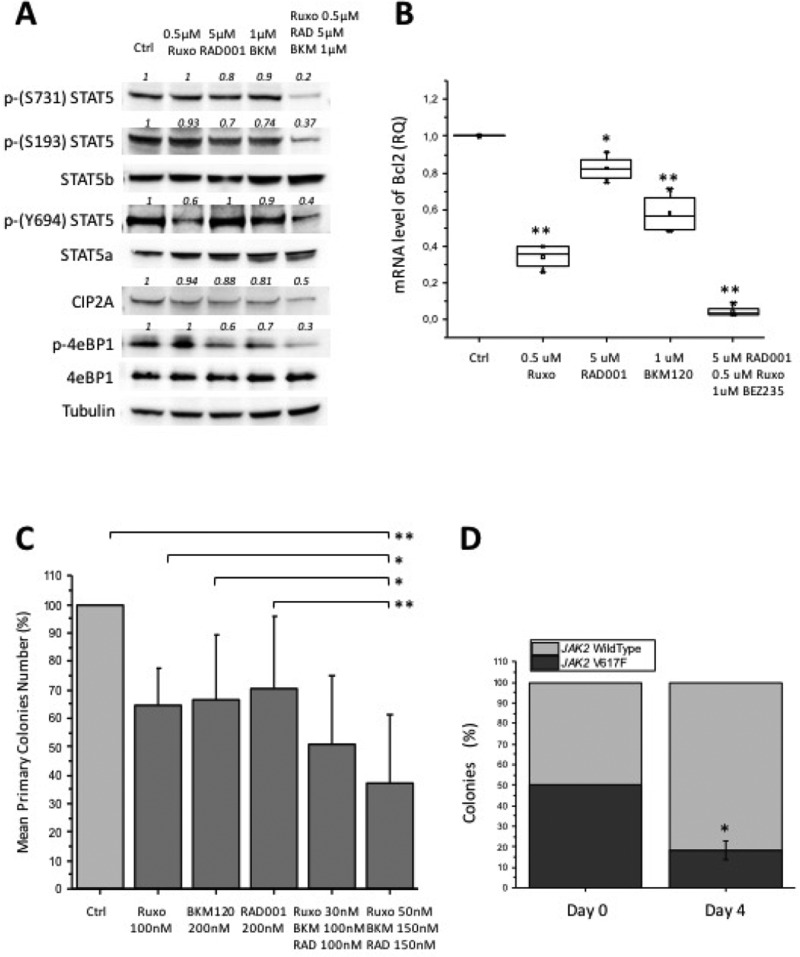
Effects of combining BKM120, RAD001 and ruxolitinib (triple drug combination) in different MPN models (**A**) SET2 cells were exposed to suboptimal amounts of BKM120, RAD001 and ruxolitinib as single drugs (see Figure 1 for comparison), or in combination, and the effects on the phosphorylation of S731, S193 and Y694 of STAT5 and of 4eBP1, and the levels of CIP2A, were assessed by western blotting. (**B**) The mRNA amount of the STAT5 target gene Bcl-2 was assessed in SET2 cells treated with single drugs or drug combination by qRT-PCR. (**C**) Bone marrow mononuclear cells isolated from 6 *JAK2*V617F patients were plated in semisolid medium in presence of single agents or in combinations at two different dose levels. Colony formation was expressed as percent of the number of colonies enumerated in control dishes (no drug). (**D**) Bone marrow cells of *JAK2*V617F KI and wild type mice were mixed in a 1:1 ratio and plated in semisolid medium in the presence of BKM120 plus RAD001 and ruxolitinib, 30 nM each. Individual colonies harvested at day 4 of culture (to avoid colonies overlapping) were genotyped by allele specific PCR. All *P* values were determined by unpaired two-tailed Student’s *T* test and confirmed by BootstRatio test on fold change data (^*^P < 0.05, ^**^P < 0.01).

Based on the above findings indicating activity of BKM120 as single agent in different MPN models, we went to combine ruxolitinib with BKM120 and RAD001. We first assessed the combination index (C.I.), to ascertain possible synergistic activity of drugs combination. The IC_50_ values were significantly lower when BKM120, ruxolitinib and RAD001 were combined compared to individual drugs, in cultures of Ba/F3 *JAK2*V617F (*P* < 0.05) and SET2 cells (*P* < 0.05) (Supplementary Table 2), indicating high synergism of triple treatment. In particular, the C.I. of triple combination of BKM120, ruxolitinib and RAD001 in SET2 was significantly lower (*P* < 0.05) than for the combination of BKM120 and ruxolitinib only. Furthermore, the combination of suboptimal doses of BKM120, RAD001 and ruxolitinib led to a stronger inhibition of the phosphorylation of tyrosine and serine residues of STAT5a and b in SET2 cells than that obtained with single drugs (Figure 5A). In order to document the downstream transcriptional consequences of reduced STAT5 activity caused by the inhibitors, we assessed the expression levels of Bcl-2, a well characterized STAT5 target gene [46], in SET2 cells exposed to single agents and triple combination. As shown in Figure 5B, Bcl-2 mRNA levels were significantly reduced by any of the drugs tested (up to a mean of 18%, 65% and 43% the baseline levels, respectively for RAD001, ruxolitinib and BKM120), while the triple combination resulted in an almost complete inhibition of gene expression (<5% the baseline levels). The triple drug combination was further assessed in clonogenic assay of hematopoietic progenitors of *JAK2*V617F mutated patients, plated in semisolid medium in presence of each single drug and two different doses of triple combination (Figure 5C). The colonies generated in presence of the most effective triple drug combination (ruxolitinib 50 nM, BKM120 150 nM, RAD001 150 nM) decreased by 62.6% compared to control cultures (*P* < 0.006), and by 33.0%, 29.3% and 27.3%, respectively, in the presence of RAD001 (*P* < 0.04), BKM120 and ruxolitinib (*P* < 0.05) as single drugs. Finally, to assess the ability of treatment to preferentially target *JAK2*V617F mutated cells, we plated BM cells obtained from *JAK2*V617F knock-in and wild-type mice [47], in a 1:1 ratio, in the presence of triple drug combination; individual colonies were harvested and genotyped. We found that triple treatment reduced the proportion of *JAK2*V617F-positive colonies from 50% at baseline to 18% on day 4 (*P* < 0.05) (Figure 5D).

### Concurrent JAK2 and PI3K and mTOR inhibition is highly effective in JAK2V617F knock-in mice

**Figure 6:**
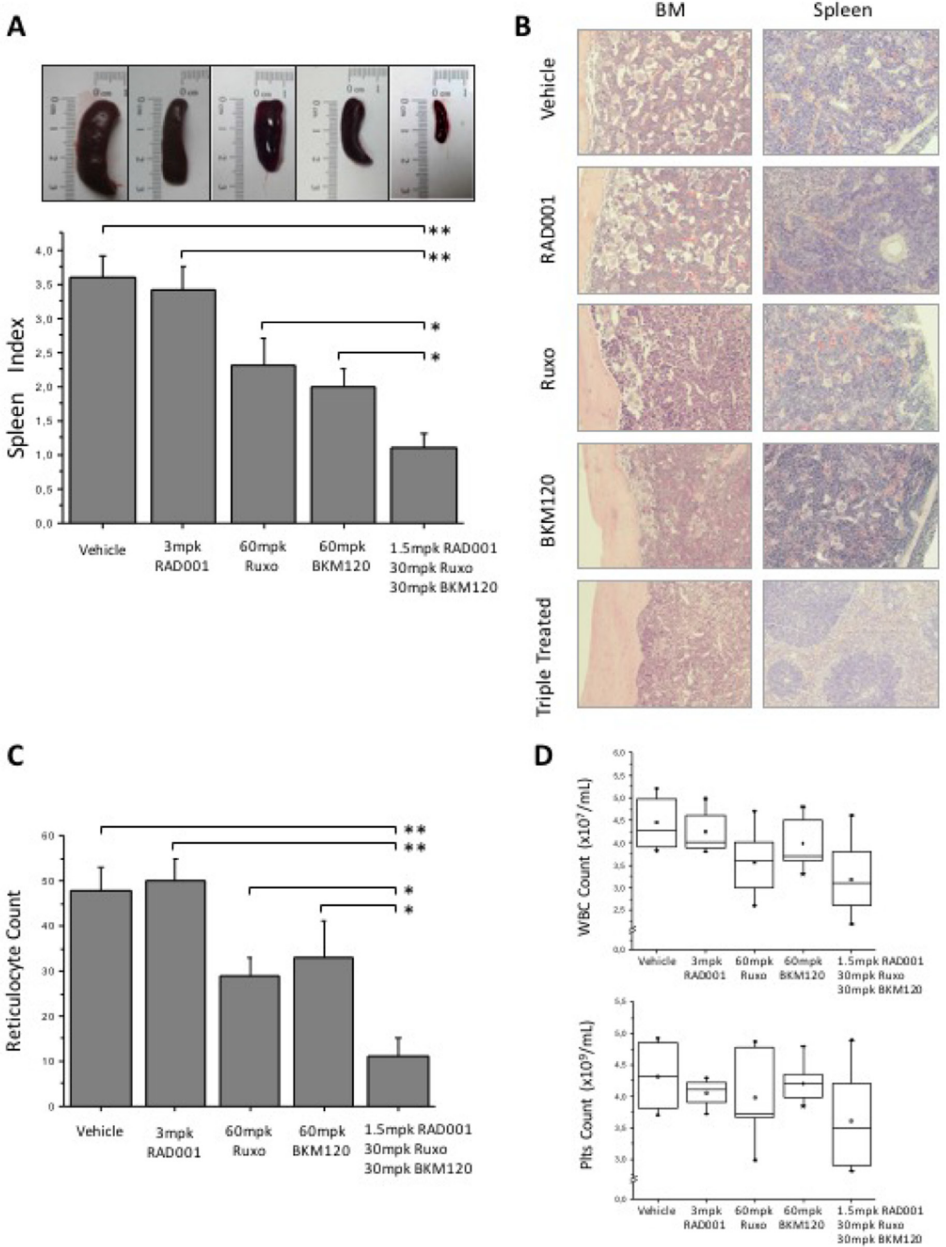
Efficacy of BKM120, RAD001 and ruxolitinib, single or in triple combination, in JAK2V617F knock-in mice Animals received treatment for 16 days before sacrifice; control mice received vehicle only with the same schedule as treated ones. 10 mice for each treatment cohort were analyzed. (**A**) Effects of triple treatment on spleen size (upper panel) and spleen index (calculated as ratio of spleen and body weight x100) compared to other treatment groups and controls. (**B**) Histopathology of the spleen and bone marrow in control and treated mice. Picture were taken with a LEICA DM LS2 microscope using a N-Plan × 20 objective. (**C**) The number of reticulocytes per 10 high power fields in peripheral blood smears. (**D**) The number of white blood cells and platelets in the peripheral blood of mice treated with drugs combination; values in mice receiving each single drug were comparable to controls and are not shown. All *P* values were determined by unpaired two-tailed Student’s *T* test and confirmed by BootstRatio test on fold change data (^*^P < 0.05, ^**^P < 0.01).

We then used *JAK2*V617F knock-in mice to test the effects of treatment with the triple combination of BKM120, RAD001 and ruxolitinib; these mice develop a progressive myeloproliferative disease starting from the first months after birth, characterized by marked erythrocytosis, thrombocytosis and leukocytosis, and splenomegaly, that mimics PV and evolves to myelofibrosis at later stages [47]. Mice were treated daily for 16 days with 3 mg/kg body weight (mpk) RAD001, 60 mpk ruxolitinib or 60 mpk BKM120, and with the combination of the three drugs at half the dose used as single agent. Treatment was well tolerated and the body weight loss was <10% in all treatment groups; histopathology of the liver revealed the absence of overt hepatotoxicity (Supplementary Figure 5). Triple drug combination induced a dramatic reduction of splenomegaly (Figure 6A), macroscopically much more evident than in animals treated with single agents. The median spleen index of triple treated cohort was 1.1 compared with 3.6 in control vehicle-treated mice (*P* < 0.01), 2, 2.3 and 3.4 respectively for BKM120, ruxolitinib (both *P* < 0.05) and RAD001 (*P* < 0.01). Histopathology showed a marked reduction of megakaryocytes and myeloid cells infiltrating the BM and the spleen in mice treated with the triple drug combination; in the spleen, the overall tissue architecture was improved towards normal appearance with splenic lymphoid follicles being restored (Figure 6B). Conversely, in mice receiving single drugs, including ruxolitinib, changes of BM and spleen cellularity and architecture were barely detected. The mean reticulocyte count per HPF in mice receiving drug combination was 11 compared to 48 in vehicle mice (*P* < 0.01), 50, 29 and 33 in mice receiving RAD001 (*P* < 0.01), ruxolitinib or BKM120 (*P* < 0.05), respectively (Figure 6C). Although not statistically significant, there was a trend towards reduced leukocyte and platelet counts in mice receiving the triple combination compared with the vehicle group (Figure 6D).

## DISCUSSION

Preventing and reducing STAT5 activation through the use of JAK2 inhibitors proved clinical efficacy in patients with myelofibrosis and PV [48, 49], and the first-in-class JAK1 and JAK2 inhibitor ruxolitinib was approved recently for selected categories of patients. In fact, constitutive activation of the JAK/STAT signaling pathway is considered the basic pathogenic mechanism of MPN, through either direct, as in patients harboring mutations of *JAK2,* or indirect, as for *MPL* or *CALR* mutations, involvement of JAK2, that in turn leads to sustained phosphorylation of STAT5. Complete deletion of STAT5a/b in *JAK2*V617F mice prevented the development of PV, thereby supporting the relevance of STAT5a/b as target for therapy [50]. However, in spite of the impressive clinical benefits in patients receiving ruxolitinib, it is still debated if the drug has the potential to modify the course of disease; in fact, the *JAK2* mutant variant allele frequency reduced appreciably in a proportion only of the patients, and complete molecular remissions were rare [51]; similarly, resolution of abnormal bone marrow histopathology was reported only occasionally, although stabilization was noted in several patients after 2–3 years of treatment [52]. One possible explanation for the partial efficacy of ruxolitinib is the persistence of STAT5 activation mediated by JAK2-dependent and/or JAK2-independent mechanisms. In previous studies, we [27, 31] and others [53, 54] showed that phosphorylation of STAT5 in cells harboring the *JAK2*V617F mutation is mediated by activated PI3K/mTOR, and could be reduced by target PI3K/mTOR inhibitors. However, the mechanistic basis of these observations, as well as the ultimate relevance for designing novel treatment strategies, remained largely unknown.

**Figure 7:**
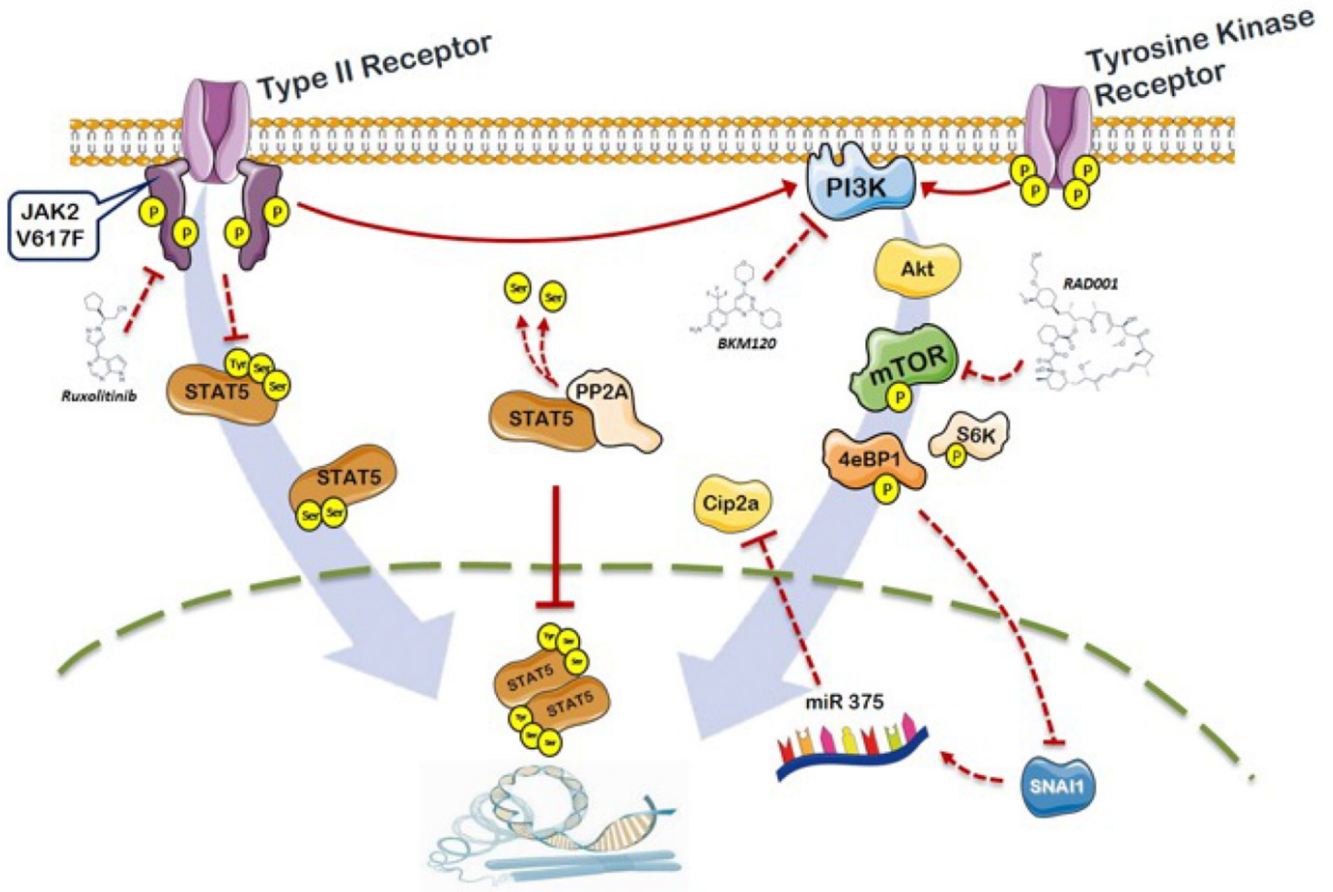
Schematic representation of the role of PI3K/mTOR signaling and PP2A/CIP2A axis in the phosphorylation of STAT5b in *JAK2*V617F mutated cells On the left part of the figure, the activated JAK2-dependent tyrosine phosphorylation of STAT5a is also depicted. Constitutive signaling by JAK2V617F promotes phosphorylation of STAT5 at the key Y694 residues; activated STAT5 monomers dimerize and translocate to the nucleus functioning as transcription factor for target genes. Ruxolitinib causes de-phosphorylation of Y694 on STAT5a, but leaves untouched the S731 and S193 residues of STAT5b that are essential for full activity of STAT5. STAT5b serine residues are targeted by PI3K/mTOR inhibitors. Cells with the *JAK2*V617F mutation express abnormally increased levels of the phosphatase 2A (PP2A) inhibitor CIP2A, that resulted down-regulated by PI3K/mTOR inhibitors *via* a microcircuit that includes SNAI1 and miR375. The JAK2/PI3K-pathway cross-talk described in this work, converging on unique phosphorylation targets on STAT5, contributes to the understanding of the pathophysiology of MPN and offers the rationale for combination therapies centered on a more effective inhibition of activated STAT5.

In this work we present evidence that MPN cells exposed to ruxolitinib, that effectively reduced phosphorylation of the key Y694 tyrosine residue of STAT5a, demonstrated persistence of phosphorylation at the serine residues of STAT5b; we also show that these residues are under the control of the PI3K/mTOR signaling and resulted specifically targeted by inhibitors acting at different levels of the pathway. We also demonstrated that the deregulated serine phosphorylation of STAT5b may be due to the defective activity of the protein phosphatase 2A as the consequence of increased inhibition exerted by the allosteric inhibitor CIP2A; furthermore, it is shown here for the first time, to the best of our knowledge, that CIP2A is over-expressed in MPN patients compared to healthy controls. The fact that CIP2A resulted down-regulated in cells exposed to different PI3K-pathway inhibitors, but not ruxolitinib, specifically linked the deregulation of PP2A/CIP2A to the PI3K/mTOR signaling in JAK2V617F mutated cells. Therefore, by relieving CIP2A inhibition of PP2A, the PI3K inhibitors would enforce PP2A action in de-phosphorylating target residues on STAT5 thereby resulting in reduced activation. The changes in CIP2A levels in cells exposed to PI3K/mTOR inhibitors were under the control of a microcircuit involving Hif1α [55, 56] and its target SNAI1 [57], and caused enhanced expression of miR-375, a known negative regulator of CIP2A transcription. A schematic representation of this pathway, on the basis of information derived from the literature and original data from this work, is illustrated in Figure 7.

These findings provide an explanation for the reported efficacy of PI3K/mTOR inhibitors in different models of MPN, as well as for the clinical efficacy of the mTOR inhibitor RAD001 in myelofibrosis, and offer a rationale for combination therapy. In fact, in our experiments, the concurrent blockade of JAK2 and PI3K/mTOR pathway signaling produced synergistic inhibition of STAT5 phosphorylation, resulting in effective prevention of cell proliferation *in-vitro* and improved control of myeloproliferation in *JAK2*V617F knock-in mice. We showed that inhibition of PI3K/mTOR pathway was best achieved by concurrent exposure to BKM120 (a PI3K inhibitor) and RAD001 (a mTOR inhibitor), that when used together might prevent the well known rebound activation of AKT by mTOR inhibitors alone. The relevance of these data for the clinics is that both drugs are already being used in clinical trials, and they might be combined as well as with low doses of ruxolitinib. Finally, our findings also suggest that PP2A may serve as a novel therapeutic target for modulating the extent of STAT5 activation in MPN.

## MATERIALS AND METHODS

### Compounds and reagents

BKM120 (a selective pan class-I PI3-Kinase inhibitor), RAD001 (a mTOR specific allosteric inhibitor active against TORC1), BEZ235 (a double PI3K and mTOR inhibitor) and ruxolitinib (a JAK1/JAK2 kinase inhibitor) were kindly provided by Novartis (Basel, Switzerland); PP242 was purchased from Sigma-Aldrich (St Louis, MO, US). *In-vitro* and *in-vivo* formulations and administration schedules are detailed in Supplementary Methods.

### Cell Lines

The following human: HEL (*JAK2*V617F mutated), K562 (BCR/Abl positive), SET2 (*JAK2*V617F mutated); and murine: Ba/F3 and Ba/F3-EPOR cells expressing *JAK2* wild-type (wt) or *JAK2*V617F (VF) cell lines were used, as previously described [27] and detailed in Supplementary Methods.

### Human and murine cells and colony assay

Peripheral blood (PB) samples were obtained from PV or PMF patients, diagnosed according to the 2008 WHO criteria, under a protocol approved by Institutional Review Board of Azienda Ospedaliera-Universitaria Careggi and after obtaining a written informed consent. Mononuclear cells (MNCs) and Granulocytes from MPN patients or control subjects were isolated by Ficoll Hipaque (Lonza). MNCs were plated at 1×10^5^/mL in methylcellulose supplemented with stimulating factors (detailed in Supplementary Methods) and enumerated on day 14 according to standard criteria. For murine colony assay, BM cells were harvested from JAK2 wild-type and V617F mice and plated in a 1:1 ratio in presence of drugs. Single colonies were harvested on day 4 and submitted to conventional PCR in order to discriminate the JAK2 mutational status.

### Confocal microscopy

Granulocytes were isolated from *JAK2*V617F MPN patients. Fixed and permeabilized cells were incubated with relevant primary antibodies followed by anti-rabbit IgG Alexa Fluor-488 conjugated. Microscopic images were taken by a LSM 510 META Zeiss confocal microscope system (Carl Zeiss Inc., Jena, Germany) and analyzed by Confocor 2 (Zeiss) software (details in Supplementary Methods).

### siRNA transfection

Gene silencing was performed by siRNAs transfection according to Nucleofector technology (Lonza): mTOR, PP1, PP2A, CIP2A and non-targeting control siRNAs were from Cell Signaling Technologies (Danvers, MA, US).

### Cell lysis and SDS-PAGE western blotting

Cells were lysed in RIPA lysis buffer containing a proteinase inhibitor cocktail and assayed in western blotting analysis, as described in Supplementary Methods. Images were acquired with ChemiDoc XRS+ (Bio-Rad, Hercules, CA, US) and analyzed with ImageJ software [58] for densitometric analysis. In all cases, a representative experiment of three is shown.

### RNA isolation and quantitative real-time PCR (qRT-PCR)

Total RNA was extracted and reverse-transcribed to cDNA, then subjected to quantitative real-time PCR (qRT-PCR) according to standard protocols. Methods and primers for qRT-PCR analysis are listed in Supplementary Methods. Relative gene expression was calculated according to the comparative cycle threshold (Ct) and 2^-DDCt^ method.

### JAK2V617F KI mouse model

All animal procedures were performed according to Italian laws in an animal facility (Ce.S.A.L., University of Florence) under humanized conditions. KI mice were generated and experimental procedures were carried out as described [31]. Three months-aged KI mice received the drugs for indicated periods administered by oral gavage and were euthanized by CO2 inhalation. Blood parameters were measured using the Sysmex XE5000 (Sysmex, Hyogo, Japan) cell counter, while reticulocytes were counted in methylene blue-stained blood smears. The spleen was collected and weighted; to accomplish for variations in body weight at baseline, a spleen index (*i.e*., spleen weight/body weight ×100) was calculated.

### Statistical methods

The level of significance from two-sided tests was *P* < 0.05. All *P* values were determined by unpaired two-tailed Student’s *T* test and confirmed by BootstRatio test [59] on fold change data. The analysis of drug synergism was performed by calculation of the combination index (CI) according to the Chou and Talaly method [60] as reported in Supplementary Methods.

## SUPPLEMENTARY MATERIALS FIGURES AND TABLES


