# Supplementary material for: Inhibitors of the PI3K/mTOR pathway prevent STAT5 phosphorylation in JAK2V617F mutated cells through PP2A/CIP2A axis
Source: Oncotarget. 2017 May 22;8(57):96710–24. doi: 10.18632/oncotarget.18073 (PMC5722516; doi:10.18632/oncotarget.18073)
Supplement: Supplementary file 1 [file oncotarget-08-96710-s001.pdf]

Inhibitors of the PI3K/mTOR pathway prevent STAT5 phosphorylation in *JAK2V617F* mutated cells through PP2A/CIP2A axis

Supplementary Materials

Reagents

BKM120 (NVP-BKM120, a selective pan class-I PI3-Kinase inhibitor), RAD001 (everolimus, a mTOR specific allosteric inhibitor with activity against mTORC1), BEZ235 (a double PI3K and mTOR inhibitor) and ruxolitinib (INCB018424, a JAK1/JAK2 kinase ATP-competitive inhibitor) were kindly provided by Novartis (Basel, Switzerland); PP242 (mTORC1/2 inhibitor) was purchased from Sigma-Aldrich (St Louis, MO, US). For in-vitro studies stock solution were prepared in dimethyl sulfoxide (DMSO; Sigma-Aldrich) to a concentration of 10 mM and further diluted to an appropriate final concentration in culture medium at the time of use. For in-vivo use, BKM120 was prepared in 10% N-methyl-2-pyrrolidone and 90% PEG300; everolimus was formulated as a microemulsion pre-concentrate, it was diluted with plain water; ruxolitinib was formulated in 0.5% hydroxypropyl methylcellulose.

RIPA lysis buffer (50 mM pH 7.4 Tris-HCl, 150 mM NaCl, 1% NP-40, 1 mM EDTA)

For *in-vitro* studies stock solution were prepared in dimethyl sulfoxide (DMSO; Sigma-Aldrich) to a concentration of 10 mM and further diluted to an appropriate final concentration in culture medium at the time of use. For *in-vivo* use, BKM120 was prepared in 10% N-methyl-2-pyrrolidone and 90% PEG300; Everolimus was formulated as a microemulsion pre-concentrate, it was diluted with plain water; was formulated in 0.5% hydroxypropyl methylcellulose. Calyculin A (Santa Cruz Biotechnologies, Santa Cruz, CA, US) was dissolved in DMSO at 0.1 mM final concentration and established concentration was delivered to cultures 2 hours prior treatment.

Cell culture

The HEL and K562 human cell lines were purchased from the German Collection of Microorganisms and Cell Cultures (DSMZ, Braunschweig, Germany). SET2 cells were obtained from ATCC (Manassas, VA, US). Murine Ba/F3 and Ba/F3-EPOR cells expressing *Jak2* wild-type or *Jak2*V617F were donated by R. Skoda (University of Basel, Switzerland). Cell lines were cultured in RPMI 1640 supplemented with 10% fetal bovine serum (FBS; Lonza, Verviers, Belgium) (20% for SET2 cells), 1% penicillin-streptomycin and 1% L-glutamine. Ba/F3 and Ba/F3-EPOR wild-type cell lines were supplemented with 10% WEHI pre-conditioned media as a source of IL-3. Recombinant human EPO (rhEPO; Sigma Aldrich) was added to *Jak2* wild-type Ba/F3-EPOR cells, that require the cytokine for survival and proliferation, at final concentration of 1 U/mL. Cells were maintained at 37C° in 5% CO2 humidified atmosphere.

Ba/F3 and Ba/F3-EPOR cells, both wild-type and V617F, K562, HEL and SET2 cells were plated at the concentration of 2×10^4 cells per mL in 96-well culture tissue plates with increasing concentrations of the drug(s), in triplicate, and the amount of viable cells was assessed at 48 h using the WST-1 assay (Roche, Basel, Switzerland) after normalization to wells containing an equivalent volume of vehicle (DMSO) only.

Primary human and murine cells and clonogenic assay

Peripheral blood (PB) or bone marrow (BM) samples were obtained from PV or PMF patients, diagnosed according to the 2008 WHO criteria, under a protocol approved by Institutional Review Board of Azienda Ospedaliera-Universitaria Careggi and after obtaining a written informed consent. Mononuclear cells (MNCs) were separated by gradient using Ficoll Hypaque (Lonza). CD34+ cells were immunomagnetically purified (Miltenyi Biotec, Gladbach, Germany) from mononuclear cells (MNCs) separated by gradient using Ficoll Hypaque (Lonza). Control CD34+ cells were obtained from discarded cord blood units. Research was carried out according to the principles of Declaration of Helsinki.

MNCs from MPN patients or control subjects were plated at 1×10^5/mL in methylcellulose (MethoCult; StemCell Technologies, Vancouver, Canada) supplemented with SCF 50 ng/mL, IL-3 10 ng/mL, IL-6 10 ng/mL, GM-CSF 10 ng/mL, G-CSF 10 ng/mL and EPO 1 U/mL for the growth of BFU-E and CFU-GM. Colonies were enumerated on day 14 according to standard criteria. For murine colony assay, BM cells were harvested from JAK2 wild-type and V617F mice and plated at $1x10^4$/mL in a 1:1 ratio in presence of drugs. Single colonies were harvested

on day 4 and submitted to conventional PCR in order to discriminate the JAK2 mutational Status.

CD34+ cells from MPNs patients or control subjects were plated at 1×10^3/mL in methylcellulose (MethoCult 4434; StemCell Technologies, Vancouver, Canada) supplemented with SCF 50 ng/mL, IL-3 10 ng/mL, IL-6 10 ng/mL, GM-CSF 10 ng/mL, G-CSF 10 ng/mL and EPO 1 U/mL for the growth of BFU-E and CFU-GM. Endogenous erythroid colonies (EEC) assay was performed by plating 2.5×10^5/ml PB mononuclear cells of PV patients in methylcellulose containing leucocyte-conditioned medium without EPO (MethoCult 4531; StemCell Technologies). For CFU-Mk, 1×10^3/ml $CD34^+$ cells were plated in Megacult Collagen medium with lipids (StemCell Technologies) supplemented with thrombopoietin 50 ng/ml, IL-3 10 ng/ml, IL-6 10 ng/ml. Colonies were counted on day 10–14 according to standard criteria.

Assessment of apoptosis and cell cycle analysis

Quantification of apoptotic cells after 48 hours drug exposure was accomplished using the Annexin-V-FLUOS Staining kit (Roche, Basel, Switzerland); at least 20,000 events were acquired in a FACS SCAN flow cytometer. For cell cycle distribution analysis, 1×10^6 cells were harvested after an incubation period of 18 hours, washed twice with PBS, fixed with ethanol 95% containing RNase 10 μg/mL and stained with propidium iodide 50 μg/mL for 15 min before flow cytometry analysis. Data were processed with Flow-Jo software (Tree Star, Ashland, OR, USA).

RNA isolation and quantitative real-time PCR (qRT-PCR)

Total RNA was extracted using TriPue Isolation Reagent (Roche Diagnostics), the RNA concentration and purity/integrity was determined with NanoDrop ND-1000 spectrophotometer (NanoDrop Techn., Wilmington, DE, USA) and reverse-transcribed to cDNA using One microgram of RNA was reverse transcribed using High Capacity cDNA Reverse Transcription Kit (Applied Biosystems, Life Technologies; Carlsbad, CA). Quantitative real-time PCR (qRT-PCR) was carried out with SYBR Green PCR master mix (Applied Biosystems) on a StepOnePlus Real-time PCR System (Applied Biosystems) according to standard protocols. The primers for the qRT-PCR analysis were as follows: for CIP2A, forward 5′-TGACCCTTCTGCTGCCTACA-3′ and reverse 5′-GCCTTGGCAATCCTTTCACA-3′; for I2PP2A, forward 5′- CGTTCGAGTCAAACGCAGAA -3′ and reverse 5′- CAGCACCTGCATCAGAATGGT -3′; for Bcl-2, forward 5′-GGACAACATCGCCCTGTG-3′ and reverse 5′-AGTCTTCAGAGACAGCCAGGA-3′; for GAPDH, forward 5′-GTCGGAGTCAACGGA-3′ and reverse 5′-GGGTGGAATCATATTGGAACATG-3′. GAPDH was used as internal control. Relative gene expression was calculated according to the comparative cycle threshold (Ct) and 2^{-DDCt} method.

Confocal microscopy

Granulocytes from *JAK2*V617F MPN patients and healthy donors were isolated by gradient using Ficoll Hypaque, resuspended in complete medium (RPMI 1640) (3×10^5 /ml) and seeded on polylysine-coated glass slides.. Cells were then incubated at 37° C and CO_2 5% and then fixed in formaldehyde (4% in PBS pH 7.2). After fixing cells were permeabilized with 0.1% Triton (Sigma) for 10 minutes, and incubated for 20 minutes at room temperature with goat serum (1 mg/ml). After, cells were incubated with antibody anti- phospho (p) STAT5 Y694 (rabbit IgG, Cell Signaling), p-STAT5 S731 (rabbit IgG, Abcam, Cambridge, UK) or p-STAT5 S193 Ab (rabbit IgG, kindly given by Prof. Francesco Annunziato from University of Florence, Italy) for 40 minutes; cell were then incubated at room temperature with anti-rabbit IgG Alexa Fluor 488 conjugated Ab for 30 minutes (2 μg/ml), in buffer containing TOPRO-3 dye (0.2 μM) for the nuclear counter staining. Cells were then washed in PBS for 5 min, and the slides mounted with Vectashield mounting medium (Vector Laboratories Inc., Burlingame CA). Microscopic images were taken by a LSM 510 META Zeiss confocal microscope system (Carl Zeiss Inc., Jena, Germany), using 40X oil immersion lens, corresponding to a 400X magnification. For images analysis Confocor 2 (Zeiss) software was used.

Cell lysis and SDS-PAGE western blotting

Cells were lysed in RIPA lysis buffer containing a proteinase inhibitor cocktail (Halt Protease Inhibitor Cocktail Kit, PIERCE, Rockford, IL, US) and assayed in western blotting analysis with the antibodies as follows: p-STAT5 Y694, mTOR, p-4eBP1 (tyrosine 70), 4eBP1 and Tubulin (Cell Signaling Technology). p-STAT5 S731, STAT5 isoform A and B (STAT5a, STAT5b) (Abcam) and p-STAT5 S193 (from Prof. Annunziato), CIP2A and PP1 were from Santa Cruz Biotechnology. PP2A was from Millipore (Merck Millipore, Darmstadt, Germania). Anti-rabbit HRP antibody was from Sigma-Aldrich and anti-mouse HRP antibody was from Millipore. Western blotting images were acquired with ChemiDoc XRS+ (Bio-Rad, Hercules, CA, US) and analyzed with ImageJ software [1] for densitometric analysis. In all cases, a representative experiment of three is shown.

Ba/F3 JAK2V617F-luc+ mouse model

All animal procedures were performed according to Italian laws in an animal facility (Ce.S.A.L., University

of Florence) under humanized conditions. Female SCID beige mice (4–6 weeks; Harlan, Indianapolis, IN, USA) were given 3 × 10^6 *JAK2*V617F-Ba/F3-EPOR luc+ cells (clone 8) 34, kindly provided by T. Radimerski (Novartis, Basel) by tail vein injection. At specified time points thereafter, mice were injected with Xeno Light D-luciferin (Caliper, Waltham, MA, USA) to generate a measurable bioluminescence signal that is proportional to luc+ cells; measurement was performed 15 min after luciferin injection using the Photon Imager apparatus (Biospace Lab, Paris, France). Baseline measurement performed on day 6 after luc+ cell injection was used to establish individual bioluminescence level, then mice were randomly divided into three treatment cohorts of six mice each having comparable baseline disease burden (vehicle, 45 mpk BKM120, 60 mpk BKM120). Drugs were administered daily by gavage. Imaging was performed at weekly intervals after the first drug dose; mice were followed daily for survival and euthanized when they developed hind limb paralysis or became moribund.

JAK2V617F KI mouse model

All animal procedures were performed according to Italian laws in an animal facility (Di.V.A.L., University of Florence) under humanized conditions. KI mice were generated and experimental procedures were carried out as previously described [2]. Three months-aged KI mice received the drugs for indicated periods administered by oral gavage and were euthanized by CO2 inhalation.

Cuts of the liver were fixed in PBS-buffered formalin (4%), paraffin embedded, sectioned and haematoxylin and eosin stained.

Statistical methods

The Mann-Whitney U or Fisher test was used for comparison using the SPSS software (version 23, StatSoft, Inc., Tulsa, OK, USA) or Origin software (v7.5, OriginLab, Northampton, MA, USA). Data were expressed as mean ± standard deviation (SD). The level of significance from two-sided Student's tests was $P < 0.05$. All P values were determined by unpaired two-tailed Student's T test and confirmed by BootstRatio test [3] on fold change data. The concentration at which 50% inhibition (IC_{50}) of cell proliferation or colony formation, promotion of apoptosis or change in distribution of the cells in cell cycle phase occurred was calculated using the Origin software. The analysis of drug synergism was performed by calculation of the combination index (C.I.), that is a measure of the interaction between two drugs. The C.I. was calculated according to the median-effect principle of the Chou and Talaly [4] method using CalcuSyn software 2.1 (BioSoft, Cambridge, UK). According to this formula, CI< 0.90, 0.90 < CI < 1.10 and CI> 1.10 were considered, respectively, synergistic, additive and antagonistic effects.

REFERENCES

1. Schneider CA, Rasband WS, Eliceiri KW. NIH Image to ImageJ: 25 years of image analysis. Nat Methods. 2012; 9:671–675.
2. Bartalucci N, Tozzi L, Bogani C, Martinelli S, Rotunno G, Villeval JL, Vannucchi AM. Co-targeting the PI3K/mTOR and JAK2 signalling pathways produces synergistic activity against myeloproliferative neoplasms. J Cell Mol Med. 2013; 17:1385–1396.
3. Cleries R, Galvez J, Espino M, Ribes J, Nunes V, de Heredia ML. BootstRatio: A web-based statistical analysis of fold-change in qPCR and RT-qPCR data using resampling methods. Comput Biol Med. 2012; 42:438–445.
4. Chou TC, Talaly P. A simple generalized equation for the analysis of multiple inhibitions of Michaelis-Menten kinetic systems. J Biol Chem. 1977; 252:6438–6442.

Supplementary Table 1: Effects of BKM120 on the colony-forming activity of progenitor cells

	BFU-E IC_{50} (nM)	CFU-GM IC_{50} (nM)	CFU-Mk IC_{50} (nM)
**Ctrl**	900 ± 370	760 ± 200	>100
**PV**	187 ± 76*	77 ± 50*	14 ± 1**
**MF**	148 ± 63*	312 ± 180**	18 ± 8**

7 patients with PMF and 10 with PV (all *JAK2*V617F mutated) and 7 healthy subjects were evaluated. Cells were plated in methylcellulose in the presence of different concentrations of the drug and the IC_{50} was calculated. The IC_{50} values were, in case of PV and PMF, respectively: 77 ± 50 nM and 312 ± 180 nM for CFU-GM (both $P < 0.05$ vs controls), 187 ± 76 nM and 148 ± 63 nM (both P<0.05) for BFU-E and 14±1nM and 18 ± 8 (both $P < 0.01$) for CFU-MK, compared with 760 ± 200 nM, 900 ± 370 nM and > 100 nM, respectively, in control subjects. All P values were determined by unpaired two-tailed Student's *T* test (* $0.01 < P < 0.05$, ** $0.001 < P < 0.01$).

Supplementary Table 2: Analysis of the effects of combination treatment on the proliferation of JAK2V617F mutated Ba/F3 and SET2 cells

Ba/F3 JAK2V617F

Single Drug IC_{50} (nM)			Combination IC_{50} (nM)			C.I.
BKM120	RAD001	Ruxo	BKM120	RAD001	Ruxo	0.25
1100 ± 207	651 ± 50	200 ± 20	299 ± 13	193 ± 7	96 ± 10	

SET2

Single Drug IC_{50} (nM)			Combination IC_{50} (nM)			C.I.
**BKM120**	RAD001	Ruxo	BKM120	RAD001	Ruxo	0.3
**1300 ± 160**	6500 ± 110	1000 ± 89	1000 ± 45	5000 ± 160	500 ± 71	

Cultures were exposed to single drugs and to drug combination and the IC_{50} value for each experimental condition was calculated. A Combination Index (C.I.) lower than 1 indicates a high synergism of co-treatment.

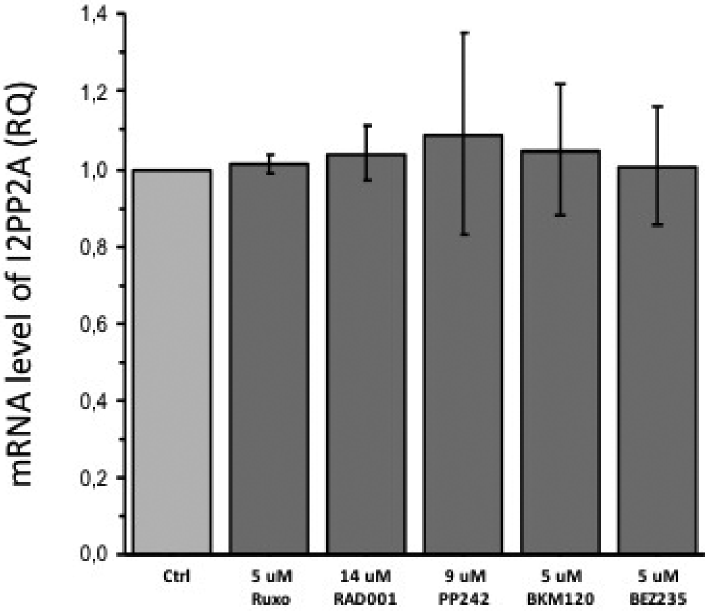

Supplementary Figure 1: I2PP2A mRNA expression is not affected by PI3K/mTOR inhibitors. Since I2PP2A is a known PP2A inhibitor, together with CIP2A, we analyzed its mRNA expression level by qRT-PCR in SET2 cells that had been exposed to PI3K-inhibitors and ruxolitinib at the same doses used for the analysis of CIP2A mRNA expression (see figure 4A for comparison). As shown in figure, none of the inhibitors used significantly modified the expression of I2PP2A compared to controls.

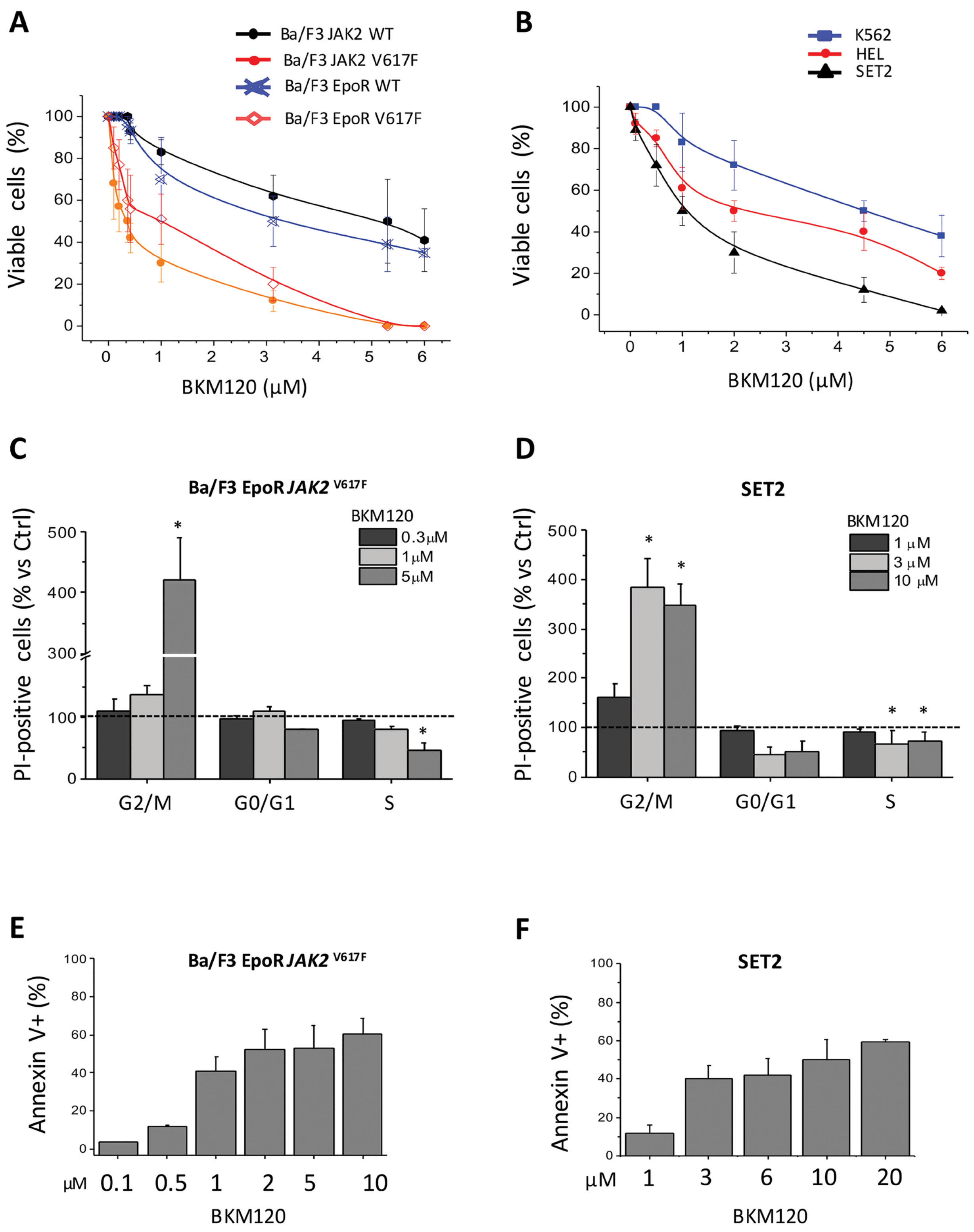

Supplementary Figure 2: The effects of the BKM120 as single agent against JAK2V617F mutated cells were evaluated using different *in-vitro* cellular models. S2A, BKM120 dose-dependently inhibited the proliferation of murine Ba/F3 cells expressing the human *JAK2*V617F mutation at concentration significantly lower (IC_{50} values, 364 ± 200 nM) than cells expressing the wild-type *JAK2* (5,300 ± 800 nM) ($P < 0.05$). Similar results were found in case of JAK2V617F-Ba/F3 cells co-expressing the EPOR: IC_{50} values were 1,100 ± 207 nM for *JAK2*V617F Ba/F3-EPOR cells and 3,122 ± 1000 nM for the wild-type counterpart ($P < 0.05$). S2B, BKM120 dose-dependent inhibition of the *JAK2*V617F-mutated human HEL and SET2 cell lines (2,000 ± 500 nM and 1,000 ± 300 nM, respectively); for comparison, the growth curve of BCR/ABL rearranged K562 cells is shown (4,500 ± 800 nM) ($P < 0.01$). S2C-D, Cell cycle analysis of *JAK2*V617F mutated Ba/F3-EpoR cells and SET2 cells; a significant increase of G2/M phase and concomitant decrease of S-phase in murine and human cell lines was documented ($P < 0.01$ versus control cells). S2E-F, BKM120 dose-dependently induced apoptosis, yet at concentrations higher than those inhibiting proliferation, in *JAK2*V617F mutated Ba/F3-EpoR cells ($IC_{50,}$ 1.8 µM) and SET2 cells ($IC_{50,}$10 µM).

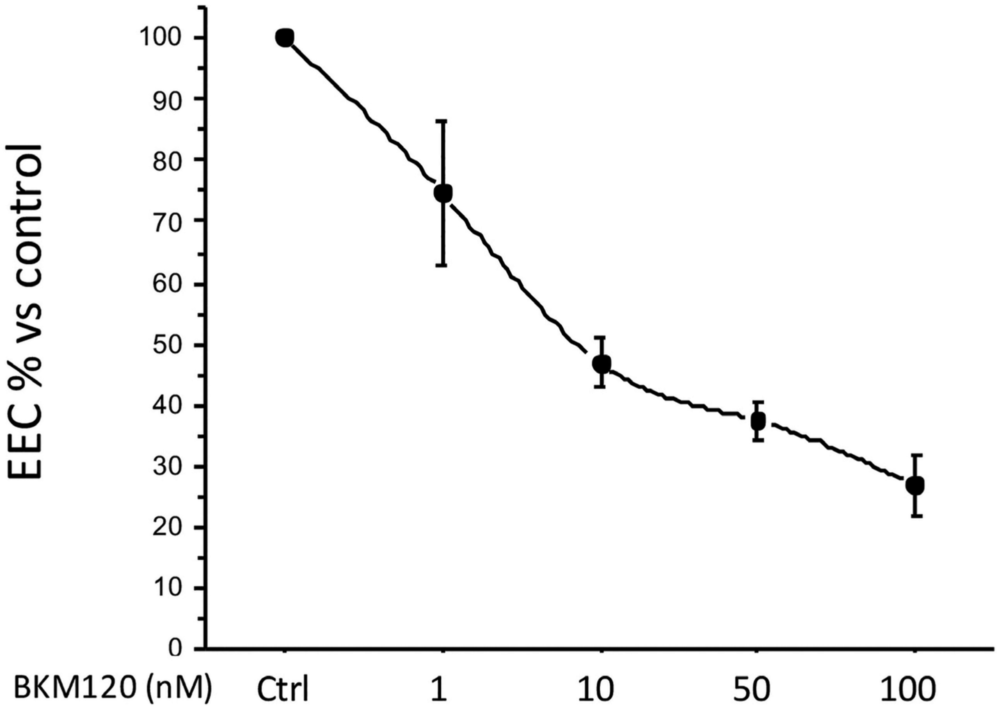

Supplementary Figure 3: BKM120 inhibits EPO-independent endogenous erythroid colonies growth. Dose-dependent inhibition of the growth of EPO-independent endogenous erythroid colonies (EEC) from patients with PV ($n = 5$) induced by BKM120; the resulting IC_{50} was 9 ± 4 nM. Unifocal hemoglobinized colonies were scored at 10 days.

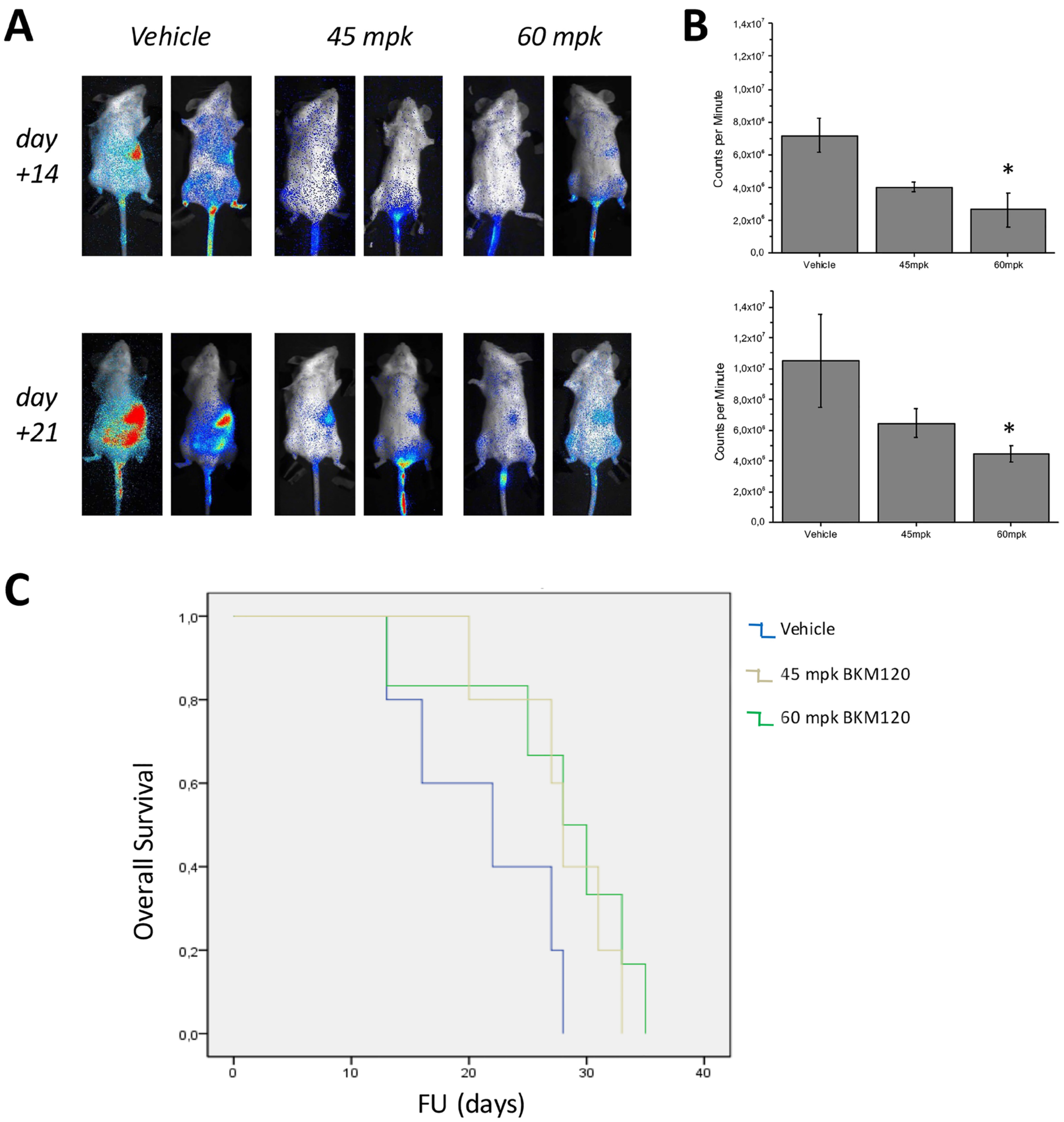

Treatment Cohort	Mean Lifespan post-Injection	Mean Lifespan post-Treatment	Mean Lifespan increment vs Veh
BKM120 60 mpk	27.8±4.9	20.8±4.9	+ 31.1 %
BKM120 45 mpk	27.3±7.8	20.3±7.8	+ 28.8 %
Vehicle	21.2±6.6	14.2±6.6	\

Supplementary Figure 4: The JAK2V617F-Ba/F3-EPOR luc+ mouse model highlights the effects of BKM120 *in-vivo*. S4A, In vehicle mice, Ba/F3 *JAK2*V617F–Luc+ cells colonize bone marrow and spleen first, followed by abdomen and the whole body, as shown by in-vivo imaging pictures taken at day 14 and day 21 after injection. Imaging revealed a lower degree of cell proliferation and dissemination in BKM120 treated mice compared to controls. S4B, The mean bioluminescence signal of different groups was quantified by counts per minute. Mice that received 60mpk BKM120 showed a signal significantly lower than 45mpk BKM120 and placebo groups ($P < 0.05$), reflecting effective inhibition of Ba/F3 JAK2V617F-Luc+ cells proliferation. S4C, Kaplan-Meier survival curves in mice receiving 60 mpk and 45 mpk of BKM120; the table reports the individual values and the percentage increment of survival compared to control group. Control animals showed a mean lifespan post-treatment of 14.2 ± 6.6 days, while mice treated with 60 mpk and 45 mpk BKM120 showed respectively a mean lifespan of 20.8 ± 4.9 and 20.3 ± 7.8 days accounting for a 31.1% and 28.8% increase of lifespan compared to untreated mice ($P < 0.05$).

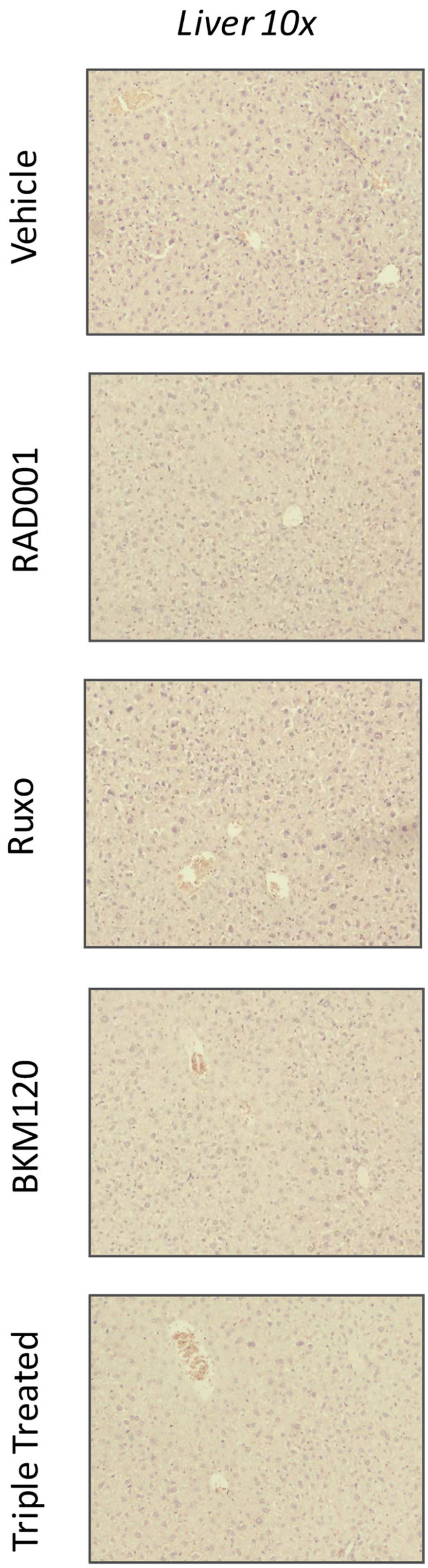

Supplementary Figure 5: Liver histopathology reveals the absence of hepatotoxicity in treated mice. Histopathology of liver tissue from mice treated with 60 mpk ruxolitinib, 3 mpk RAD001, 60 mpk BKM120, the combination of the three drugs at half the dose used as single agent and vehicle. There was no morphological evidence of hepatotoxicity with the treatment employed.
